# Hysteretic temperature dependence of resistance controlled by gate voltage in LaAlO_3_/SrTiO_3_ heterointerface electron system

**DOI:** 10.1038/s41598-022-10425-3

**Published:** 2022-04-19

**Authors:** Yongsu Kwak, Woojoo Han, Joon Sung Lee, Jonghyun Song, Jinhee Kim

**Affiliations:** 1 Korea Research Institute of Standards and Science, Daejeon, 34113 South Korea; 2 Department of Physics, Chungnam National University, Daejeon, 34134 South Korea; 3 Department of Nanoscience, University of Science and Technology, Daejeon, 34113 South Korea; 4 Display and Semiconductor Physics, Korea University Sejong Campus, Sejong, 30019 South Korea; 5 Institute of Quantum Systems (IQS), Chungnam National University, Daejeon, 34134 South Korea

**Keywords:** Electronic properties and materials, Surfaces, interfaces and thin films

## Abstract

For two-dimensional electron gas device applications, it is important to understand how electrical-transport properties are controlled by gate voltage. Here, we report gate voltage-controllable hysteresis in the resistance–temperature characteristics of two-dimensional electron gas at LaAlO_3_/SrTiO_3_ heterointerface. Electron channels made of the LaAlO_3_/SrTiO_3_ heterointerface showed hysteretic resistance–temperature behavior: the measured resistance was significantly higher during upward temperature sweeps in thermal cycling tests. Such hysteretic behavior was observed only after application of positive back-gate voltages below 50 K in the thermal cycle, and the magnitude of hysteresis increased with the applied back-gate voltage. To explain this gate-controlled resistance hysteresis, we propose a mechanism based on electron trapping at impurity sites, in conjunction with the strong temperature-dependent dielectric constant of the SrTiO_3_ substrate. Our model explains well the observed gate-controlled hysteresis of the resistance–temperature characteristics, and the mechanism should be also applicable to other SrTiO_3_-based oxide systems, paving the way to applications of oxide heterostructures to electronic devices.

## Introduction

Since the discovery of a two-dimensional electron gas (2DEG) at the all-oxide LaAlO_3_ (LAO)/SrTiO_3_ (STO) heterointerface and the ensuing exploration for its various novel properties such as superconductivity^1^, ferromagnetism^2,3^ and strong spin–orbit coupling^4^, there has been a flurry of study to uncover the origin of such peculiar characteristics and their correlations. The efforts have been paid for by inventions of new devices; the LAO/STO heterostructure is as a strong candidate for novel application in electronics because the discovered properties are gate-tunable. For example, superconductor-to-insulator transition, phase diagram similar to those of high-temperature superconductors^5^ and Lifshitz transition^6^ have been observed via back-gate biasing across the gate insulator single crystal STO that has a high dielectric constant. The gate-controllability of physical properties via STO substrate was also shown in other STO-based 2DEG systems such as γ-Al_2_O_3_/STO^7^, LAO/La_1-x_Sr_x_MnO_3_/STO^8^, CaZrO_3_/STO^9^. In addition, the two-dimensional property of electrical channels in those systems allows the devices to be significantly reduced in thickness, which is another advantage of these 2DEG systems^10^.

The band insulator STO used as the substrate of LAO/STO heterostructure is one of the key elements for the formation of a 2DEG. According to the polar catastrophe scenario, whether the interface has *n*-type or *p*-type carriers after electronic reconstruction is determined by the termination of the STO substrate surface^11^. Experimentally, 2DEGs (two-dimensional hole gases, 2DHGs) showing *n*-type (*p*-type) properties were found at the interface between LAO and TiO_2_ (SrO) terminated STO^12,13^. Additionally, oxygen vacancies, strain, and the structural phase transition of STO have been reported to affect the properties of the 2DEG^14–19^. A notable peculiarity in properties of STO is that the bulk STO undergoes a structural phase transition from cubic to tetragonal at 105 K. Kalisky et al. observed that the domain structure, attributed to the direction of TiO_6_ octahedral rotation via the phase transition, caused an inhomogeneous electrical transport showing locally enhanced conductivity near the domain boundaries^17^. Because the domain boundaries were formed randomly after each cubic-to-tetragonal phase transition, electrical transport was sensitive to thermal cycling above the structural phase transition temperature^2,17^.

The domain boundary effect was especially pronounced in patterned devices with dimensions comparable with or smaller than the typical domain size. Anisotropic electrical resistance was observed in the patterned devices with domain walls inside, and there was a hysteresis in the temperature *versus* resistance loop measured during the cool-down and warm-up processes^18^. The hysteresis of electrical resistance was reported to be maximized in nanoscaled devices^19^. However, considering the fact that the domain structure will remain the same when the temperature is cycled within the temperature range below the phase transition point (*T* ~ 105 K)^17^, the domain wall mechanism suggested in the previous studies does not fully explain the observed hysteric behavior in resistance.

Here, we investigate the hysteretic behavior in the temperature-dependent resistance (*R*(*T*)) of a microscale Hall bar-patterned LAO/STO device. It is found that application of gate voltages causes hysteresis in the *R*(*T*) loop, and the magnitude of hysteresis increases with the applied positive gate voltage. To explain the observed *R*(*T*) loop, we introduce a model that relates the *R*(*T*) hysteresis to the temperature-dependent dielectric constant of STO, and to electron trapping within the STO substrate.

## Results and discussion

**Figure 1:**
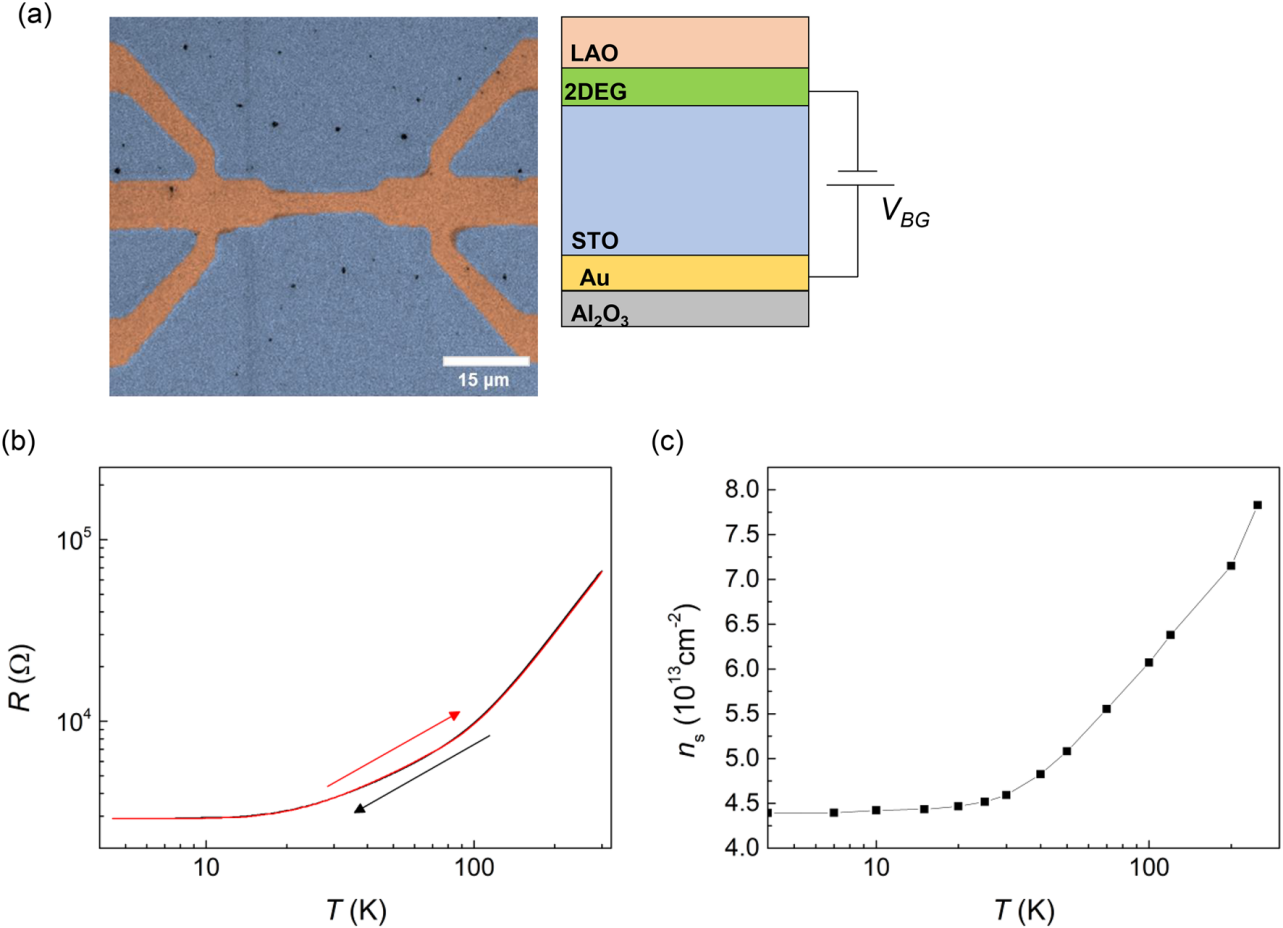
(**a**) SEM image of the Hall bar-patterned device. Inset shows schematic device cross section with a metallic back-gate electrode. In gating experiments, *V*_*BG*_ was applied between Au and 2DEG electrodes. (**b**) Temperature dependence of resistance for cool-down and warm-up processes with no *V*_*BG*_ applied. (**c**) Temperature-dependent carrier density for the cool-down process with *V*_*BG*_ = 0 V.

Figure 1a shows a top-view SEM image and a cross-sectional schematic of the device. The hall bar-patterned device was fabricated by using photolithography and Ar-ion milling on a LAO/STO sample grown by pulsed laser deposition. After the fabrication, the back-gate electrode was prepared by directly attaching a gold film deposited on an Al_2_O_3_ single crystal plate to the bottom surface of the STO substrate using a silver paste. Therefore, as shown in Fig. 1a, the Au electrode was sandwiched between the bottom of the STO substrate and Al_2_O_3_ single crystal, and was used to apply back-gate voltage (*V*_*BG*_) to investigate the gate dependence of resistance. The Al_2_O_3_ plate was used for electrical insulation.

Figure 1b shows the inherent temperature dependence of resistance with no back-gate voltage applied. The resistance shows metallically decreasing behavior with lowering temperature, and there is no hysteresis in *R*(*T*) between the cool-down and the warm-up processes. This ensures that our experimental instruments, such as the temperature control system and the gate voltage source, do not cause any problem that may result in false hysteresis or anomalies in *R*(*T*). Figure 1c shows temperature dependence of the carrier density for *V*_*BG*_ = 0 V. The carrier density is changed from 8 × 10^13^ cm^−2^ to 4.5 × 10^13^ cm^−2^ with lowering temperature. At *T* = 4 K, the carrier density is higher than 1 × 10^13^ cm^−2^, a value known as the Lifshitz transition point^6^.

**Figure 2:**
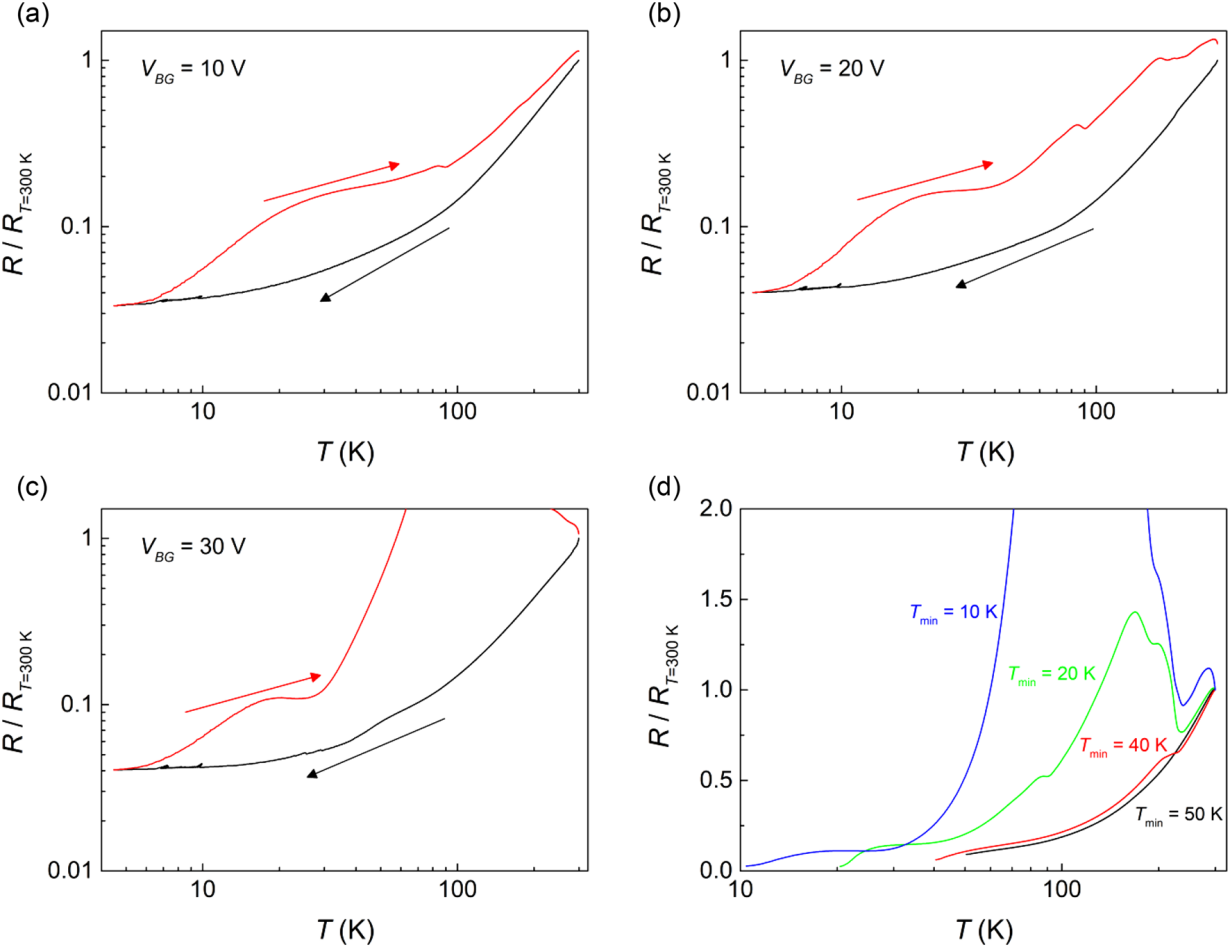
Temperature dependence of the normalized resistance (*R*(*T*)/*R*_*T* = 300 K_) for cool-down and warm-up processes with (**a**) *V*_*BG*_ = 10 V, (**b**) *V*_*BG*_ = 20 V, (**c**) *V*_*BG*_ = 30 V. Black and red curves show *R*(*T*)/*R*_*T* = 300 K_ for cool down and warm-up processes, respectively. (**d**) *R*(*T*)/*R*_*T* = 300 K_ for several warm-up process at *V*_*BG*_ = 30 V with various *T*_*min*_, where *T*_*min*_ is the target base temperature for the preceding cool-down sequence from room temperature.

To investigate if a resistance hysteresis occurs when *V*_*BG*_ is applied, *R*(*T*) for both cool-down and warm-up processes were measured. Figure 2a–c show the temperature dependence of the normalized resistance (*R*(*T*)/*R*_*T* = 300 K_) measured under different applied *V*_*BG*_. During the cool-down processes, *R*(*T*)/*R*_*T* = 300 K_ shows metallic property for all *V*_*BG*_, similar to that for *V*_*BG*_ = 0 V. On the other hand, during the warm-up processes, anomalous *R*(*T*)/*R*_*T* = 300 K_ showing hysteric behavior are observed. In the lowest temperature range, that is, while the temperature is warmed from 4 to 6 K, *R*(*T*)/*R*_*T* = 300 K_ are similar to those obtained during the cool-down. However, the *R*(*T*)/*R*_*T* = 300 K_ curves start to deviate from the respective cool-down curves above 6 K, and the deviation intensifies with increasing temperature: the values of resistance are higher than those measured during the cool-down. Furthermore, the *R*(*T*)/*R*_*T* = 300 K_ for the warm-up processes show some common features which can be summarized as follows. First, when the applied *V*_*BG*_ is higher, the difference in the normalized resistance between the cool-down and warm-up processes becomes larger. Second, smooth decreases of the slope of *R*(*T*)/*R*_*T* = 300 K_ curve are observed from *T* = 20 K during the warm-up, and sudden decreases immediately followed by recoveries in resistance are observed at a higher temperature around *T* = 80 K. Third, even though there is a hysteresis in *R*(*T*)/*R*_*T* = 300 K_, the values of resistance near room temperature approach a single value (~ 10^5^ Ω) irrespective of the process history.

Figure 2d shows the dependence of *R*(*T*)/*R*_*T* = 300 K_ on the minimum temperature (*T*_*min*_) measured for several warm-up processes with a common *V*_*BG*_ = 30 V, where *T*_*min*_ is the target base temperature to which the device is cooled down from room temperature. To obtain the data in Fig. 2d, the temperature was lowered to *T*_*min*_, then *R*(*T*) was measured while increasing the sample temperature from *T*_*min*_ to room temperature. The same experimental procedure was repeated for several different *T*_*min*_. As shown in Fig. 2d, R(*T*)/*R*_*T* = 300 K_ with *T*_*min*_ = 50 K is qualitatively similar to that obtained with *V*_*BG*_ = 0 V (Fig. 1b). On the other hand, when *T*_*min*_ is below ~ 40 K, strongly increased *R*(*T*)/*R*_*T* = 300 K_ is observed for warm-up sequences, and the magnitude of hysteresis increases with decreasing *T*_*min*_.

Experimental results similar to that shown in Fig. 2 have been reported not only for LAO/STO^19^ but also for SrNbO_3_/STO^20^, while we also have observed a similar *R*(*T*) hysteresis from STO/LAO/STO trilayer (see Fig. S1 in supplementary). These previous studies argue that the observed resistance hysteresis is originated from the domain walls created by the cubic-to-tetragonal structural phase transition of STO at *T* = 105 K. Furthermore, it is also claimed that the formation of domain wall network at low temperatures causes the 2DEG to change from metal to insulator with increasing temperature, only for the devices with nanoscale channel width^19^. If the domain wall structure is an important factor of the *R*(*T*) hysteresis, it should be observed randomly^18^ and also should be sensitive to thermal cycling, when the electron channel of the LAO/STO device is laterally confined within the typical domain size. However, the hysteresis in *R*(*T*) was always observed whenever we applied positive *V*_*BG*_ on the device and lowered the sample temperature below 50 K. Considering these observations, especially the influence of *V*_*BG*_ on the hysteresis, we propose that the dielectric characteristic of STO, rather than domain walls, is the main origin of the hysteretic behavior of *R*(*T*). This idea is also supported by the fact that *T*_*min*_ being less than 50 K is essential for the hysteresis to occur, as shown in Fig. 2d. If the domain wall were the main cause of the resistance hysteresis, we should have observed a small or little dependence of hysteresis on *T*_*min*_, owing to the fact that the structural phase transition of STO occurs at *T* = 105 K.

**Figure 3:**
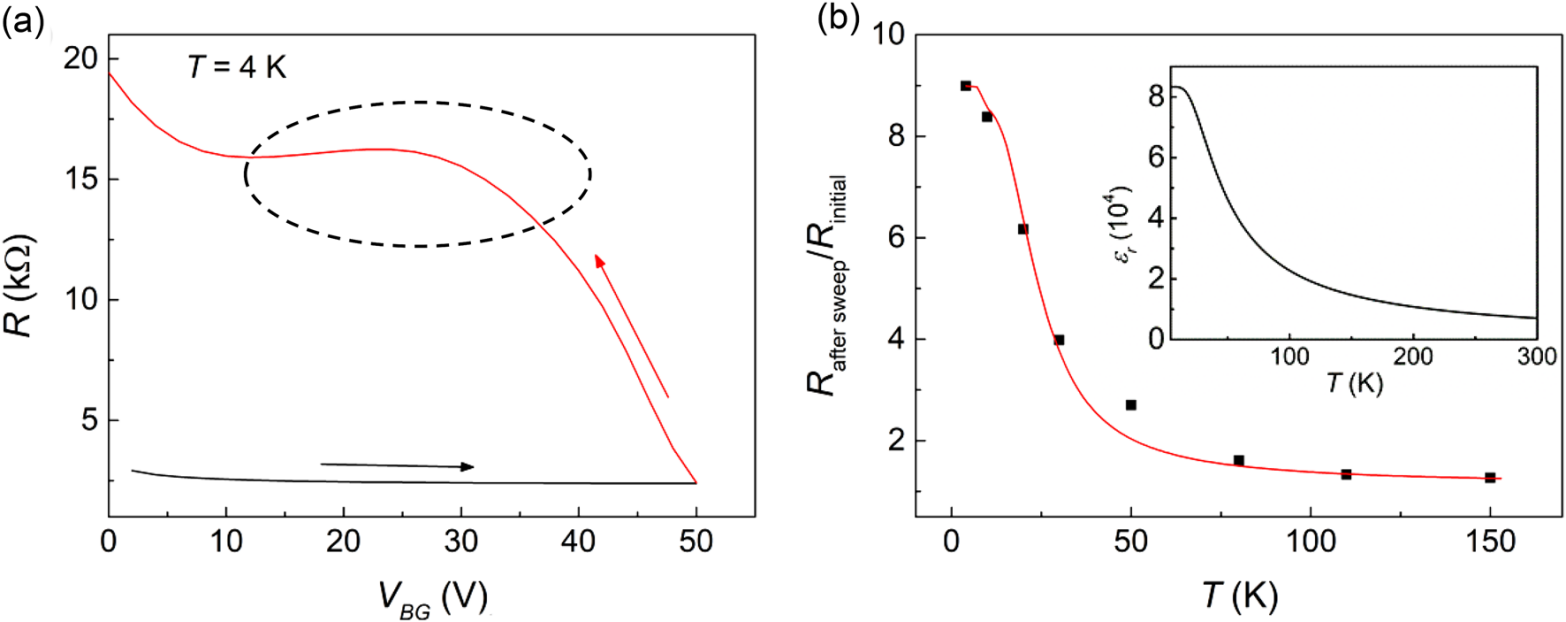
(**a**) *V*_*BG*_ dependence of the resistance at 4 K. Black (red) line shows the gate voltage-dependent resistance with increasing (decreasing) *V*_*BG*_ from 0 V (50 V) to 50 V (0 V). (**b**) Black squares represent the ratio of resistance after gate sweeping at *V*_*BG*_ = 50 V (*R*_after sweep_) to resistance without any gate voltage (*R*_initial_). The red line is a curve fitted based on the revised empirical formula. The temperature-dependent dielectric constant in the inset (obtained from Barrett's formula)^30^ and *V*_*BG*_ = 50 V were used for the curve fitting. We obtained the following parameters for a 95% confidence interval: *α* = 200 ± 35 V, *β* = 0.9 ± 0.18, *n*_0_ = (1.3 ± 0.17) × 10^14^ cm^−2^.

To figure out the detailed effect of *V*_*BG*_ at the lowest temperature, which is the turning point of the thermal cycling processes in Fig. 2a–c, we measured the sample resistance while sweeping *V*_*BG*_ in forward and backward directions at *T* = 4 K. Here, the forward sweep (backward sweep) was carried out by changing *V*_*BG*_ from 0 V (50 V) to 50 V (0 V). Figure 3a shows the *V*_*BG*_ dependence of resistance, which is strongly hysteretic: the measured resistance was much larger for the backward sweep than for the forward sweep. As is indicated by a dotted circle in Fig. 3a for the backward sweep of *V*_*BG*_, the increasing trend of resistance with decreasing *V*_*BG*_ subsides and *R*(*V*_*BG*_) forms a depression between *V*_*BG*_ = 30 V and 10 V. We should note that this retracing curve of *R*(*V*_*BG*_) is similar to the shape of *R*(*T*)/*R*_*T* = 300 K_ observed near *T* = 20 K during the warm-up process in Fig. 2. This suggests that the observed hysteresis of *R*(*T*)/*R*_*T* = 300 K_ in Fig. 2 is closely related to the hysteretic behavior of resistance shown in Fig. 3a, which took place just by sweeping *V*_*BG*_ to and back from a positive value.

To investigate the relationship between the hysteresis in the *R*(*T*) and *R*(*V*_*BG*_) in Figs. 2 and 3, we used a planar capacitor model^21^ where the electron density injected by applying *V*_*BG*_ is described as *ε*_0_*ε*_*r*_*V*_*BG*_*/ed*, where *ε*_0_ is the vacuum permittivity, *ε*_*r*_ is the relative permittivity of STO, *e* is the electron charge, and *d* is the thickness of the STO substrate. Considering that the dielectric constant *ε*_*r*_ of STO increases drastically at low temperatures, it can be seen that sweeping down the temperature under a fixed *V*_*BG*_ plays a role that is similar to a *V*_*BG*_ sweep with regard to the injected electron density. Thus, it can be deduced that the hysteretic *R*(*T*) phenomenologically arises from the same origin with that for the hysteretic *R*(*V*_*BG*_) at a fixed temperature. Therefore, it is necessary to figure out the origin of hysteresis in the *R*(*V*_*BG*_) to understand the hysteresis of *R*(*T*).

It has been reported by Yin et al. that the gate-dependent resistance of LAO/STO shows a similar hysteretic behavior, which has been attributed to electron trapping that results in a difference in the electron density of 2DEG between the forward and backward *V*_*BG*_ sweeps^22^. When *V*_*BG*_ is swept, the amount of total injected electrons during the forward sweep should be same with the amount of removed electrons during the backward sweep. During the forward sweep, electrons are injected into the 2DEG channel and the impurity sites as well. In the backward sweep, on the other hand, electrons are removed only from the 2DEG because the electrons trapped at the impurity sites are energetically more stable. As a result, the electron density of 2DEG after a *V*_*BG*_ sweep is decreased as much as the trapped electron density (*n*_*tr*_). In that report, the trapped electron density is described by an empirical formula, *n*_*tr*_ = *n*_0_{1 − exp(− *V*_*BG*_/*α*)}, where *n*_0_ is the maximum electron density that can be trapped at the impurity sites, and *α* is a fitting parameter for each sample. However, it is not possible to correlate the hysteresis in *R*(*T*) to the trapped electrons using that empirical formula, because the influence of the dielectric constant of STO substrate is not factored in. Since the injected electron density in the planar capacitor model is proportional to the electric displacement, which is dependent not only on *V*_*BG*_ but also on the temperature via *ε*_*r*_(*T*), we modified the above formula into *n*_*tr*_ = *n*_0_{1 − exp(− *ε*_*r*_(*T*)*V*_*BG*_/*ε*_*r*_(0)*α*)}, which becomes practically identical to the original formula when the temperature is fixed 0 K. The use of *ε*_*r*_(0) instead of *ε*_*r*_(*T*_*min*_) is just for simplicity; any difference between the two values will be compensated by the fitting parameter *α*.

To confirm the reliability of this revised formula for description of electron trapping at impurity sites, we fit the temperature dependence of the resistance ratio, that is *R*_after sweep_/*R*_initial_, where *R*_after sweep_ is the resistance measured after a *V*_*BG*_ sweep up to 50 V and *R*_initial_ is that measured before the application of *V*_*BG*_. Considering that the electron density of 2DEG decreases to (*n*_*in*_ − *n*_*tr*_) after a *V*_*BG*_ sweep due to the electron trapping, *R*_after sweep_/*R*_initial_ equals *μ*_*in*_*n*_*in*_/{*μ*_after sweep_(*n*_*in*_ − *n*_*tr*_)} according to the Drude formula (*R* = 1/*neμ*), where *n*_*in*_, *μ*_*in*_, and *μ*_after sweep_ are the initial electron density, the initial mobility, and the mobility after the *V*_*BG*_ sweep of the 2DEG, respectively. Assuming that *μ*_after sweep_ = *μ*_in_*β*, where *β* is another fitting parameter and *n*_*tr*_ is described by the revised empirical formula, the measured *R*_after sweep_/*R*_initial_ is fit by using the parameters: *α* = 200 V, *β* = 0.9, and *n*_0_ = 1.3 × 10^14^ cm^−2^. Figure 3b shows the temperature dependence of *R*_after sweep_/*R*_initial_ along with the fitted curve. The conformity of the fitting result demonstrates that the revised empirical formula correctly reflects the effect of electron trapping at the impurity sites throughout the experimental temperature range. This also indicates that the hysteretic *R*(*T*) is owing to the electron trapping caused by application of *V*_*BG*_, along with the strongly temperature-dependent dielectric property of STO^23^.

**Figure 4:**
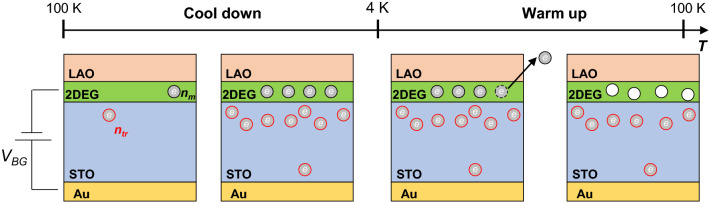
Schematic of our model for the *R*(*T*) hysteresis. During the cool-down process, the temperature-dependent dielectric constant of STO makes *n*_*tr*_ and *n*_*m*_ to be increased even though a fixed positive *V*_*BG*_ is applied throughout. For the warm-up process, the electrons that have been injected into the 2DEG, as well as preexisting electrons, are removed prior to the electrons injected and trapped at impurity sites, owing to the said temperature dependence of dielectric constant.

Now we present an explanation for the observed hysteresis in *R*(*T*). When a positive gate voltage is applied, electrons are injected not only into the 2DEG but also into the impurity sites, as is explained above. The electron density injected into the impurity sites (*n*_*tr*_) is described by the revised empirical formula with the constraint of *n*_*m*_ + *n*_*tr*_ = *ε*_0_*ε*_*r*_*V*_*BG*_*/d* from the planar capacitor model^21^, where *n*_*m*_ is the injected electron density in the 2DEG and thus stays itinerant. When the device is cooled down with an applied gate voltage, increasing dielectric constant of the STO with lowering temperature causes *n*_*m*_ and *n*_*tr*_ to be increased (see Fig. 4). This does not change the metallic property of *R*(*T*) for the cool-down process. On the other hand, when the device is being warmed up after the cool-down, the injected electrons should be removed due to the decreased dielectric constant of the STO with increasing temperature. However, because the electrons trapped at the impurity sites are energetically stable, the itinerant electrons at the 2DEG are removed prior to the trapped electrons, as in the case of the *V*_*BG*_ sweep in Fig. 3a. As a result, there occurs a difference in the electron density of 2DEG between the cool-down and warm-up processes, which produces the observed resistance hysteresis. At higher temperatures (*T* ≥ 250 K), the resistance values measured during the warm-up process approach those measured during the cool-down, as a greater part of the trapped electrons can now escape due to the thermal effect^22,24^.

The above scenario provides a good explanation of the *V*_*BG*_ dependence of the observed resistance hysteresis intensity shown in Fig. 2a–c. It also rationalizes well the *T*_min_ dependence of resistance hysteresis shown in Fig. 2d, in the light of the dielectric constant of STO that increases with decreasing temperature especially rapidly below 50 K. Since the temperature dependence of the dielectric constant of STO is the key factor of the *R*(*T*) hysteresis, similar gate-dependent *R*(*T*) hysteresis is expected to occur commonly in STO-based 2DEG devices.

As a final remark, we comment on the differences and improvements of our model from preexisting explanations for the resistance hysteresis in LAO/STO. Some of previous studies pointed out the domain boundary structure as the main cause of the resistance hysteresis^18–20^ However, the data shown in Fig. 2 reveals that the *R*(*T*) hysteresis starts to develop from as low as 6 K, which is much lower than the structural phase transition temperature 105 K. Our model provides a good rationale of this experimental observation. There have been a number of experimental studies on the *V*_*BG*_-dependent hysteretic resistance at fixed temperatures that attribute the gate hysteresis to electron trapping^22,24–29^. In this work, we introduce the dramatically strong temperature dependence of dielectric constant of the quantum paraelectric STO into the picture of gate hysteresis by electron trapping, and develop a model that successfully reproduces the observed *R*(*T*) hysteresis. We further infer that the channel width dependence of *R*(*T*) hysteresis reported previously^19^ may be explained partly in terms of stronger focusing of the gating field near a narrower channel.

## Conclusion

We studied gate voltage-controllable hysteresis in the resistance–temperature characteristics of two-dimensional electron gas at LaAlO_3_/SrTiO_3_ heterointerface. Without an applied gate voltage, the *R*(*T*) curves measured during cool-down and warm-up processes were almost the same. However, a hysteresis in *R*(*T*) was manifest after applying a gate voltage, and its intensity was increased by increasing the applied gate voltage *V*_*BG*_. The magnitude of the hysteresis also increased with lowering the base temperature of the cool-down process. These observations of resistance hysteresis were explained in terms of electron injection into the 2DEG conduction channel at the LAO/STO heterointerface and electron trapping at the impurity sites of STO substrate. In the presented scenario, the strongly temperature-dependent dielectric constant of STO plays an important role. We believe that our model of gate-dependent *R*(*T*) hysteresis can also be applied to interpretation of electrical transport in other STO-based devices, and may lead to better control over the device properties.

## Methods

### LaAlO_3_ thin films growth

Before deposition of LAO, TiO_2_-terminated STO was annealed to obtain an atomically flat terrace on the STO surface at 950 °C under oxygen partial pressure of 2 × 10^–5^ Torr for two hours. The LAO thin film of 8 unit-cells was deposited on the TiO_2_-terminated STO by using pulsed laser deposition at 750 °C under oxygen partial pressure of 10^–5^ Torr. For the deposition, a KrF excimer laser with an energy of 120 mJ and a repetition rate of 4 Hz was used. After the LAO layer deposition, the sample was maintained in an oxygen partial pressure of 500 mTorr at 750 °C for 30 min and cooled down from 750 °C to room temperature.

### Electrical transport measurements

*R*(*T*) was measured during cool-down from room temperature to minimum temperature (*T*_*min*_) and the subsequent warm-up. The temperature was changed at a rate of 3 K/min, while applying a back-gate voltage to the electrode coated on the back-side surface of STO. The temperature was controlled by Quantum Design PPMS, and the sample resistance was measured using standard lock-in techniques.

## Supplementary Information

Supplementary Figure S1.
