# Supplementary material for: Hysteretic temperature dependence of resistance controlled by gate voltage in LaAlO3/SrTiO3 heterointerface electron system
Source: Sci Rep. 2022 Apr 19;12:6458. doi: 10.1038/s41598-022-10425-3 (PMC9019089; doi:10.1038/s41598-022-10425-3)
Supplement: Supplementary file 1 — Supplementary Figure S1. [file 41598_2022_10425_MOESM1_ESM.docx]

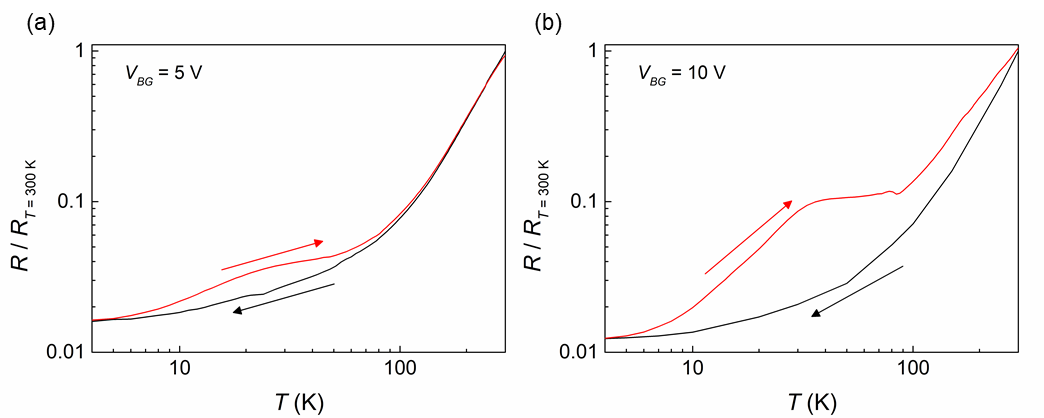


**Figure S1** (a) Temperature dependence of normalized resistance (*R*/*R_T_* _= 300 K_) for cool-down and warm-up processes with (a) *V_BG_* = 5 V (b) *V_BG_* = 10 V in STO/LAO/STO trilayer. Black and red curves show *R*(*T*)/*R_T_* _= 300 K_ for cool down and warm-up processes, respectively.
